# Phosphoproteomic Analysis Reveals Rio1-Related Protein Phosphorylation Changes in Response to UV Irradiation in *Sulfolobus islandicus* REY15A

**DOI:** 10.3389/fmicb.2020.586025

**Published:** 2020-12-03

**Authors:** Qihong Huang, Zijia Lin, Pengju Wu, Jinfeng Ni, Yulong Shen

**Affiliations:** CRISPR and Archaea Biology Research Center, State Key Laboratory of Microbial Technology, Microbial Technology Institute, Shandong University, Qingdao, China

**Keywords:** archaea, *Sulfolobus islandicus*, phosphoproteome, UV irradiation, Rio, protein phosphorylation

## Abstract

DNA damage response (DDR) in eukaryotes is largely regulated by protein phosphorylation. In archaea, many proteins are phosphorylated, however, it is unclear how the cells respond to DNA damage through global protein phosphorylation. We previously found that Δ*rio1*, a Rio1 kinase homolog deletion strain of *Sulfolobus islandicus* REY15A, was sensitive to UV irradiation. In this study, we showed that Δ*rio1* grew faster than the wild type. Quantitative phosphoproteomic analysis of the wild type and Δ*rio1*, untreated and irradiated with UV irradiation, revealed 562 phosphorylated sites (with a Ser/Thr/Tyr ratio of 65.3%/23.8%/10.9%) of 333 proteins in total. The phosphorylation levels of 35 sites of 30 proteins changed with >1.3-fold in the wild type strain upon UV irradiation. Interestingly, more than half of the UV-induced changes in the wild type did not occur in the Δ*rio1* strain, which were mainly associated with proteins synthesis and turnover. In addition, a protein kinase and several transcriptional regulators were differentially phosphorylated after UV treatment, and some of the changes were dependent on Rio1. Finally, many proteins involved in various cellular metabolisms exhibited Riol-related and UV-independent phosphorylation changes. Our results suggest that Rio1 is involved in the regulation of protein recycling and signal transduction in response to UV irradiation, and plays regulatory roles in multiple cellular processes in *S. islandicus*.

## Introduction

Protein post-translational modifications (PTMs) are rapid regulatory mechanisms of cells in response to various stresses. In eukaryotes, protein phosphorylation, one of the most important PTMs, mediates multiple cellular processes and signal cascades, including DNA damage response (DDR) and DNA repair (Sirbu and Cortez, 2013; Jette and Lees-Miller, 2015; Blackford and Jackson, 2017).

Transcriptomic studies shown that multiple genes exhibited transcriptional changes in several archaea after UV or γ-irradiation, including the up-regulation of genes involved in DNA repair, and the down-regulation of genes involved in DNA replication and cell division (McCready et al., 2005; Frols et al., 2007; Gotz et al., 2007; Williams et al., 2007). Phosphoproteomic analyses showed that a number of archaeal DDR and DNA repair proteins were regulated by phosphorylation (Aivaliotis et al., 2009; Esser et al., 2012; Reimann et al., 2013). Importantly, some kinases were induced or repressed by DNA damages. For example, *Saccharolobus solfataricus* Rio1, an atypical protein kinase, was transcriptionally induced at the early stage (30 min) of UV-treatment and the mRNA level of Rio1 increased in γ-irradiated *Pyrococcus furiosus* cells (Gotz et al., 2007; Williams et al., 2007). Another study showed that a typical (Hanks-type) protein kinase (homolog of SiRe_2030) was repressed in UV-treated *S. solfataricus* (Frols et al., 2007). In addition, two regulators, Orc1-2 and TFB3, were found to be phosphorylated in *S. solfataricus* and *Sulfolobus acidocaldarius* (Esser et al., 2012; Reimann et al., 2013). These observations suggest that protein phosphorylation plays an important role in DDR and/or DNA repair. However, a detailed mechanism of how these processes are regulated is still unknown.

Rio kinases are found in all three domains of life. In eukaryotes, Rio kinases participate in ribosome biogenesis, cell cycle progression, and genome integrity (Angermayr et al., 2002; Ferreira-Cerca et al., 2012; Widmann et al., 2012). Importantly, human RIO1 is overexpressed in different tumor types and promotes tumor growth (Weinberg et al., 2017). Recently, yeast Rio1 interactome containing more than 100 putative interactors has been mapped (Iacovella et al., 2018). Yeast Rio1 was able to regulate the transcription of a number of genes by binding to their promoter regions (Iacovella et al., 2018), indicating that Rio1 plays a global regulatory role in the cell. Although archaeal Rios was suggested to have a conserved role in ribosome biogenesis (Knuppel et al., 2018), their function has not been established thus far. Our previous study revealed that Rio1 phosphorylated the Holliday junction resolvase Hjc in *S. islandicus* and regulated its nuclease activity (Huang et al., 2019). However, it remains unclear what other role(s) archaeal Rio1 plays in DNA damage-induced regulatory networks. And above this, the effects of DNA damage on the entire phosphoproteome have not been reported in archaea.

Mass spectrometry-based phosphoproteomics is a very powerful tool for identifying and quantifying protein phosphorylation, and has been widely applied in the investigation of eukaryotic DDR and DNA repair (Ajon et al., 2011; Makarova et al., 2015; van Wolferen et al., 2016; Cao et al., 2019). In this study, we performed quantitative phosphoproteomic analysis on *S. islandicus* REY15A and *rio1* deletion strain treated or untreated with UV. We found that both UV-treatment and *rio1* deletion resulted in phosphorylation changes of proteins in various cellular processes. Moreover, we found that the phosphorylation changes in Δ*rio1* were much fewer than those in E233S after UV irradiation, most of which were related to protein metabolism. The UV-independent phosphorylation regulated by Rio1 was also analyzed, indicating that phosphorylation changes of many proteins participate in various cellular metabolic processes, reminiscent of those in yeast.

## Materials and Methods

### Strains and Growth Conditions

*Sulfolobus islandicus* strain REY15A (E233S) (Δ*pyrEF*Δ*lacS*) (hereafter E233S) and its transformants were cultured as described previously (Deng et al., 2009) with slight modification. In particular, yeast extract was not added to the growth medium. D-arabinose (0.2% w/v) was used for the induction of protein overexpression. Gelrite (0.8% w/v) was used for making culture plates.

### UV Sensitivity Assay

Assay of the sensitivity of strains to UV irradiation on plates was performed as described previously (Huang et al., 2019). For UV-treatment of cells in liquid medium, a 30 mL aliquot at an OD_600_ ∼ 0.2–0.3 was transferred to a petri dish (9 cm in diameter). The cells were irradiated with UV at 100 or 200 J/m^2^ (Frols et al., 2007) in a CL-1000 UV crosslinker (UVP Bio-imaging systems, CA, United States) or mock-treated, and transferred back to the culture flasks. The cells were then incubated at 75°C in the dark. The OD_600_ values were measured every 6 or 12 h. Growth curves were drawn based on data from three biological repeats.

### Microscopy Analysis and Colony Formation Assay

The wild type and Δ*rio1* strains were cultivated at an initial OD_600_ ∼ 0.04. After growing overnight, the samples (OD_600_ ∼ 0.2) were taken and observed under a Nikon Eclipse80i Microscope (Nikon corporation, Japan). The sizes of the cells (in diameter) were measured with software NIS-Elements AR 3.1. Samples at different times from early to late log phase were also taken and diluted by 10,000-fold, and 2–10 μl of the diluted samples were plated in triplicate. The plates were incubated at 75°C for 7–10 days before colony counting.

### Sample Preparation for Phosphoproteomic Analysis

A volume of 250 mL of the wild type or Δ*rio1* (Δ*sire_0171*) strain was cultivated to OD_600_ ∼ 0.28. A 50 mL aliquot of each culture was then transferred to petri dish (15 cm in diameter) and put into a CL-1000 Ultraviolet crosslinker. The cells were irradiated with UV at 200 J/m^2^ or mock-treated and then transferred back to a culture flask. The cells were then incubated at 75°C for 30 min with shaking at 110 rpm in the dark before collected by centrifugation at 6,245 *g* for 10 min and washed with PBS (phosphate-buffered saline) buffer three times. The cell pellets were stored at −80°C before phosphoproteomics analysis. Each sample was prepared in duplicate. The sample treatment and proteomic and bioinformatic analysis were conducted by BPI (Beijing Protein Innovation Co., Ltd, Beijing, China).

### Cell Lysis and Protein Digestion

Samples were first ground in liquid nitrogen into a powder and then transferred to a 5 ml centrifuge tube. After that, four volumes (500 μl) of lysis buffer (10 mM dithiothreitol, 1% protease inhibitor cocktail (Merck, Darmstadt, Germany), and 1% phosphatase inhibitor) was added to the cell powder, followed by sonication three times on ice using a high intensity ultrasonic processor (Scientz, Ningbo Xinzhi Ultra-Sonic Technology Co., LTD., Zhejiang, China). An equal volume of Tris-saturated phenol (pH 8.0) was then added, and the mixture was further vortexed for 5 min. After centrifugation (4°C, 5,500 *g*, 10 min), the upper layer of the phenol phase was transferred to a new centrifuge tube. Proteins were precipitated by adding five volumes (2 mL) of ammonium acetate-saturated methanol, and the samples were then incubated at −20°C overnight. After centrifugation at 5,500 *g* for 10 min at 4°C, the supernatant was discarded. The remaining precipitate was washed with ice-cold methanol once, followed by ice-cold acetone three times. The proteins were re-dissolved in 8M urea, and the protein concentration was determined with a BCA (Bicinchoninic acid) Protein Assay kit (Thermo Fisher Scientific, Waltham, MA, United States) according to the manufacturer’s instructions.

For digestion, the protein solutions were reduced with 5 mM dithiothreitol for 30 min at 56°C and then alkylated with 11 mM iodoacetamide for 15 min at room temperature in the dark. The urea concentration of the protein sample was then diluted by adding 100 mM triethylamonium bicarbonate (TEAB) to less than 2 M. Finally, trypsin was added at 1:50 trypsin to protein mass ratio for the first digestion at 37°C overnight and a 1:100 trypsin to protein mass ratio for a second 4 h digestion.

### Peptide Labeling and Enrichment

After trypsin digestion, peptides were desalted using a Strata X C18 SPE column (Phenomenex, Torrance, CA, United States) and vacuum dried. Peptides were then resuspended in 0.5 M TEAB and processed according to the manufacturer’s protocol for the TMT kit. Briefly, one unit of TMT reagent was thawed and dissolved in 100% acetonitrile. The peptide mixtures were then incubated at room temperature for 2 h and pooled, desalted, and dried by vacuum centrifugation using an Eppendorf Concentrator plus.

The tryptic peptides were then fractionated using high pH reverse-phase HPLC on a Thermo Agilent 300Extend C18 column (5 μm particles, 4.6 mm inner diameter, 250 mm length). Briefly, peptides were first separated with a gradient of 8−32% acetonitrile (pH 9.0) over 60 min into 60 fractions. Then, the peptides were combined into four fractions and dried by vacuum centrifugation for 6 h.

Tryptic peptide mixtures were incubated with an IMAC (immobilized metal affinity chromatography) microsphere suspension with vibration in loading buffer (50% acetonitrile/6% trifluoroacetic acid) and enriched phosphopeptides were then collected by centrifugation. To remove non-specifically adsorbed peptides, the IMAC microspheres were sequentially washed with 50% acetonitrile/6% trifluoroacetic acid and 30% acetonitrile/0.1% trifluoroacetic acid. Elution buffer containing 10% NH_4_OH was added, and the enriched phosphopeptides were eluted from the IMAC microspheres by vibration. The supernatant containing phosphopeptides was collected and lyophilized by vacuum centrifugation. The samples were desalted according to the instruction of the C18 ZipTips column for LC-MS/MS analysis.

### LC-MS/MS Analysis

Tryptic peptides were dissolved in 0.1% formic acid (solvent A) and then directly loaded on a homemade reverse-phase analytical column (15 cm length, 75 μm inner diameter). Solvent A contained 0.1% formic acid in 2% acetonitrile. The gradient comprised a range of 4–22% solvent B (0.1% formic acid in 90% acetonitrile) over 40 min, 23–35% over 8 min, climbing to 80% in 4 min then holding at 80% for the last 4 min, all at a constant flow rate of 400 nL/min on an EASY-nLC 1000 UPLC system.

These peptides were subjected to an NSI source followed by tandem mass spectrometry (MS/MS) on a Q Exactive^TM^ Plus (Thermo Fisher Scientific, Waltham, MA, United States) coupled inline to the UPLC. The electrospray voltage applied was 2.0 kV. The m/z scan range was 350−1800 for the full scan, and intact peptides were detected by the Orbitrap at a resolution of 70,000. The fixed first mass was set as 100 m/z. Peptides were selected for MS/MS using an NCE setting of 28, and the fragments were detected on the Orbitrap at a resolution of 35,000. A data-dependent procedure that alternated between one MS scan followed by 10 MS/MS scans with 30.0 s dynamic exclusion was then applied. The automatic gain control (AGC) was set to 1E5. The signal threshold was set as 20,000 ions/s, and the maximal injection time was set as 100 ms.

The resulting MS/MS data were processed using the Maxquant search engine (v.1.5.2.8). Tandem mass spectra were then searched against the *Sulfolobus* uniprot database (Uniprot_*Sulfolobus_islandicus*_REY15A, 3993 sequences). A reverse decoy database was concatenated for calculation of the false positive rate (FPR). Trypsin/P was specified as the cleavage enzyme allowing up to 2 missing cleavages. The minimum length of peptides was set to 7 aa, and the maximum number of modification sites was set to 5. The mass tolerance for precursor ions was set to 20 ppm in the first search and 5 ppm in the main search, and the mass tolerance for fragment ions was set as 0.02 Da. Carbamidomethyl on cystine was specified as a fixed modification. Phosphorylation on Ser/Thr/Tyr was specified as variable modifications. The quantitative method was set as TMT-10plex. Protein identification and the FDR of the identified peptide spectrum match (PSM) were adjusted to <1%. The localization probability of site modifications was filtered with a value of >0.75.

### Bioinformatic Analysis

The Gene Ontology (GO) annotation proteome was derived from the UniProt-GOA database^1^. First, the identified protein IDs were converted to UniProt IDs and then mapped to GO IDs. If some identified proteins were not annotated by UniProt-GOA database, InterProScan software was used to annotate the protein’s GO function based on the protein sequence alignment method. Then proteins were classified by Gene Ontology annotation based on three categories: biological process, cellular component, and molecular function. Functional descriptions of the identified protein domains were annotated by InterProScan v.5.14-53.0^2^ based on the protein sequence alignment method, and the InterPro domain database was used. The Kyoto Encyclopedia of Genes and Genomes (KEGG) database was used to annotate protein pathways in this dataset. The KEGG online service tool KAAS was used to annotate each protein’s KEGG database description. Then the annotation results were mapped on the KEGG pathway database using the KEGG online service tools KEGG mapper.

Soft MoMo (motif-x algorithm) was used to search for a motif of sequences derived from amino acids in specific positions of modified-13-mers (6 amino acids upstream and downstream of a site) in all protein sequences. All the database protein sequences were used as the background database parameter. The minimum number of occurrences was set to 20. The emulate original motif-x option was set, and all other parameters were set to the default.

To perform enrichment analysis of GO, KEGG, and protein domain for each category, a two-tailed Fisher’s exact test was employed to test the enrichment of each differentially modified protein against all identified proteins. The GO/pathway/protein domains with a corrected *p*-value < 0.05 were considered significant.

### Plasmid Construction

*Sulfolobus islandicus* FHA and vWAs were co-expressed in *E. coli* using the pRSFDuet-1 plasmid (Novagen). The genes encoding FHA and vWAs were amplified with corresponding primers (Supplementary Table S1) and inserted into the NdeI/XhoI and SacI (or BamHI for the vWA2 gene)/SalI, respectively. For co-expression of FHA and vWAs in *S. islandicus*, the arabinose promoter was cloned into the original multiple cloning site (MCS) of pSeSD (Peng et al., 2012). The gene fragments of Flag-vWAs and His-FHA were amplified and inserted into the XhoI/NheI in MCS1 and NdeI/SalI in MCS2, respectively, of the modified pSeSD, yielding pSeSD-Flag-vWA1-His-FHA and pSeSD-Flag-vWA2-His-FHA.

The vector pET15b was applied for the construction of plasmids expressing N-terminal His-tagged Sis7d (SiRe_0668) and SMC-like protein (SiRe_0508) in *E. coli*. The genes were amplified using their corresponding primers (Supplementary Table S1). The PCR products were digested with NdeI/SalI and ligated into the same restriction sites in pET15b. The phosphor-mimic mutants, Sis7d-T41E/S47E and SiRe_0508-Y263E/Y266E were constructed by SOE PCR and inserted into the NdeI/SalI sites of pET15b.

### Protein Purification

The plasmids for protein expression were transformed into *E. coli* BL21-Codonplus-RIL. *E. coli* harboring the pET15b plasmids was cultivated in 1 L of LB medium containing ampicillin (100 μg/ml) and chloramphenicol (34 μg/ml) (LAC). Proteins were induced by addition of IPTG and incubation at 37°C for 4 h. The cells were collected by centrifugation at 10,000 *g* for 3 min and disrupted by sonication in lysis buffer (50 mM Tris-HCl pH 8.0, 200 mM NaCl, 5% glycerol). The proteins were purified with Ni-NTA agarose (Invitrogen) column, pooled and frozen by liquid nitrogen for storage at −80°C.

### Pull Down Assay

To detect the interaction between *S. islandicus* FHA and vWAs in *E. coli*, pRSFDuet-1 based co-expression plasmids were transformed into *E. coli* BL21-Codonplus-RIL. Single transformants were picked into 30 ml LAC medium and subsequently inoculated in 500 ml LAC medium. His-tagged FHA and Flag-tagged-vWAs were induced as described above and purified by anti-Flag beads (Sigma) according the standard protocol. Briefly, *E. coli* cells were resuspended in 40 ml buffer A (50 mM Tris-HCl pH 7.5, 150 mM NaCl, 5% glycerol) and cell extract was obtained by sonication. The supernatant of heat-treated cell extract was incubated with anti-Flag beads pre-equilibrated with buffer A at 25°C for 1 h. The beads were washed with 9 ml of buffer A and the target proteins were eluted with 3 ml of 0.1 M Gly-HCl buffer. The eluted samples were analyzed by Western blot using anti-His antibody to detect the presence or absence of His-vWAs. pRSFDuet-1 containing the single gene (either *FHA* or *vWA*) was used as negative control. Similar procedure was followed for the pull down assay of the interaction between FHA and vWAs.

To analyze the interaction between FHA and vWAs *in vivo*, pSeSD based co-expression plasmids were transformed into *S. islandicus*. One hundreds milliliters of *S. islandicus* cells harboring the co-expression plasmid were cultivated to OD600∼0.2 and the protein expression was induced with 0.2% D-arabinose for 12 h. The cells were collected and resuspended in 10 ml lysis buffer and cell extract was obtained by sonication. Soluble His-tagged FHA was purified with Ni-NTA agarose column and eluted with 2 ml lysis buffer containing 250 mM imidazole. The eluted samples were analyzed by Western blot using anti-Flag antibody to detect the presence or absence of Flag-vWAs. Strains harboring the expression plasmids for single gene, pSeSD-His-FHA, pSeSD-Flag-vWA1, or pSeSD-Flag-vWA2, were used as negative controls. Similar procedure was followed for the pull down assay of the interaction between FHA and vWAs from *S. islandicus* REY15A.

### Electrophoretic Mobility Shift Assay (EMSA)

Electrophoretic mobility shift assay was performed in a 20 μl reaction mixture containing 25 mM Tris-HCl pH 8.0, 25 mM NaCl, 5 mM MgCl_2_, 1 mM DTT, 5% glycerol, FAM-labeled dsDNA (500 bp), and various concentrations of the target protein. The mixture was incubated at 37°C for 30 min and analyzed in 8% native PAGE. The gel was scanned by TyphoonTM 162 FLA 7000 image analyzer (GE Healthcare).

## Results and Discussion

### *Δrio1* Grew Faster Than the Wild Type Under Physiological Conditions

**FIGURE 1:**
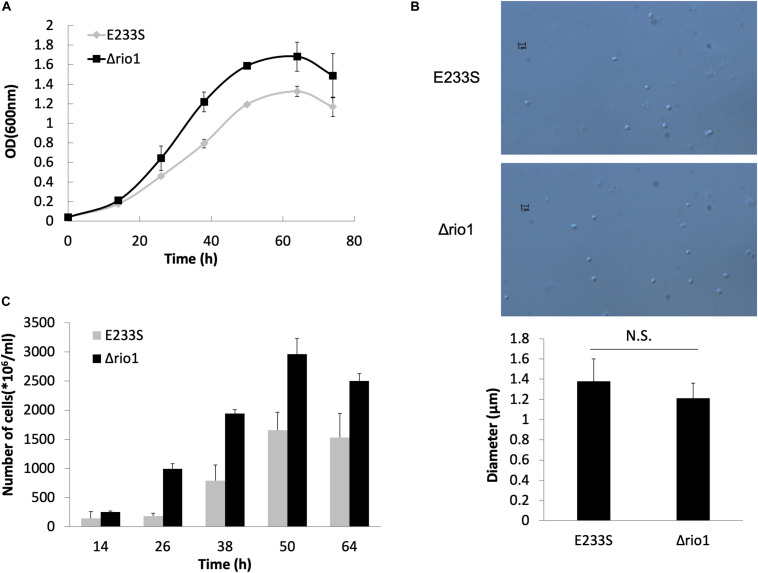
Δ*rio1* grew faster than the wild type strain E233S. **(A)** Growth curves of Δ*rio1* and E233S. The cultures were transferred to fresh medium at a same initial OD_600_ value. Samples were taken at an interval of 6 or 12 h for measuring ODs until stationary phase. **(B)** Cell size of Δ*rio1* and E233S. The samples at early log phase (OD_600_ ∼ 0.2) were used for microscopy. Diameters of 190–200 cells were measured for each strain and the averages are shown at the bottom. Representative photos are shown. Scale bar (2 μm) is indicated. N.S., non-significant. **(C)** Colony formation assay of Δ*rio1* and E233S. Samples of the two strains were taken at same time point during their growth and diluted for plating. Colonies were counted after 7–10 days incubation at 75°C.

Previous transcriptomic analysis in *S. solfataricus* revealed that *rio1*, a kinase gene, was up-regulated by 2.8-fold upon UV irradiation (Gotz et al., 2007), and the *rio1* deletion strain (Δ*rio1*) of *S. islandicus* exhibited higher sensitivity to UV irradiation than the wild type strain E233S (Huang et al., 2019). Here, we confirmed that Δ*rio1* was more sensitive to a relatively high dosage of UV irradiation than E233S. In liquid medium, both E233S and Δ*rio1* were sensitive to UV irradiation, but Δ*rio1* was slightly more sensitive to UV irradiation at 200 J/m^2^ than E233S (Supplementary Figure S1A). In the spotting assay, Δ*rio1* grew almost the same as the wild type after UV irradiation at 15 J/m^2^ on plates, but it grew more slowly than the wild type at 25 J/m^2^ UV (Supplementary Figure S1B). Interestingly, in liquid culture Δ*rio1* grew faster (5.3 h/generation) and reached a higher density than E233S (6.3 h/generation) with or without UV treatment (Figure 1A and Supplementary Figure S1A). The mean cell size of Δ*rio1* (1.21 ± 0.15 μm) was comparable to that of the wild type (1.38 ± 0.22 μm) (Figure 1B). Colony formation assay revealed that Δ*rio1* contained more live cells than the wild type (Figure 1C). The results indicate that Rio1 negatively regulates cell growth under normal growth conditions.

### Experimental Design for Phosphoproteomic Analysis and Data Reliability

**FIGURE 2:**
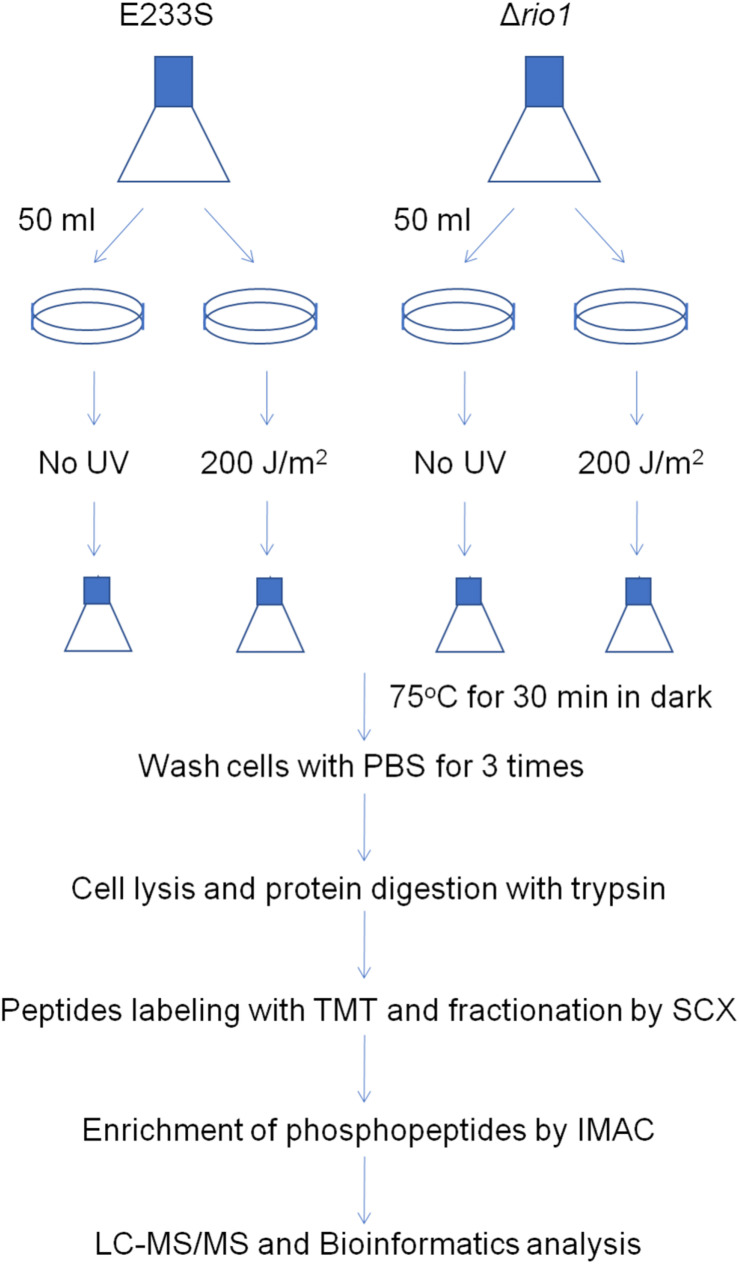
Schematic of sample preparation for phosphoproteomics analysis. A volume of 250 mL of E233S and Δ*rio1* strain was cultivated to OD_600_ ∼ 0.28. Aliquots of 50 mL were transferred to petri dishes (15 cm in diameter), which were subsequently put in a CL-1000 Ultraviolet crosslinker. Cells were irradiated with 200 J/m^2^ UV or mock-treated and transferred back to a culture flask. The flasks were incubated at 75°C for 30 min in the dark. Cells were collected by centrifugation at 6,245 *g* for 10 min and washed with PBS buffer three times. The cell pellets were ground in liquid nitrogen, and total proteins were digested with trypsin. The peptides were labeled with TMT and fractionated by SCX. The phosphopeptides were enriched by IMAC and subjected to LC-MS/MS and bioinformatics analysis. Two repeats were used for each treatment.

To reveal the phosphorylation regulatory network in DDR and Rio1-mediated processes in *S. islandicus*, we performed quantitative phosphoproteomic analysis on both UV-treated and untreated wild type strain E233S and Δ*rio1*. A schematic of the experimental design is shown in Figure 2. E233S was untreated or treated with UV to investigate the UV phospho-signaling changes. The samples of untreated and UV-treated Δ*rio1* were compared to determine whether the UV-induced differential phosphorylation events observed in wild type cells were a result of Rio1 signaling. Comparison between E233S and Δ*rio1* was performed to reveal Rio1-mediated phosphorylation changes. In a previous transcriptomic analysis (Gotz et al., 2007), *S. solfataricus* (a close relative of *S. islandicus*) cells were treated with 200 J/m^2^ UV. Therefore, the same dosage of UV was applied in the analysis in this study. The samples were then incubated for 30 min in the dark after UV irradiation before collection for quantitative phosphoproteomic analysis by TMT labeling. Two biological replicates in the early exponential growth phase (OD_600_ of 0.28) were analyzed for each sample, and relative standard deviation (RSD) and hierarchical clustering analyses of all differential phosphorylation evens (>1.3-fold, see bellow, Cao et al., 2019) from five comparison data confirmed that these measurements were reliable (Supplementary Figures S2A,B).

### Overview of Phosphoproteome of *S. islandicus* REY15A

Overall, 1357 peptides were identified, containing 672 phosphorylated sites from 352 proteins. Among these phosphorylated sites, 562 phosphorylated sites from 333 proteins were identified with localization probabilities > 0.75, and 448 unique phosphorylated sites from 284 proteins were further quantified (Supplementary Table S1). A previous phosphoproteomic analysis of *S. solfataricus* upon change of carbon source identified 690 phosphorylated sites from 540 proteins (Esser et al., 2012), while another phosphoproteomic analysis of two protein phosphatase deletion strains (Δ*pp2a* and Δ*ptp*) in *S. acidocaldarius* showed that numbers of phosphorylated sites increased compared with those from a wild type strain (from 54 to 477 and 715, respectively) (Reimann et al., 2013). Our data are comparable to those from these reports. Among the proteins identified in our study, more than 61% of the phosphorylated proteins contained only one phosphorylated site, while the proteins with two and three phosphorylated sites were 19.0 and 10.2%, respectively. Fewer than 10% of the phosphorylated proteins contained more than three phosphorylated sites (Supplementary Figure S3). Specifically, there were 13 proteins containing five or more phosphorylated sites, indicating those proteins were intensively regulated by phosphorylation. These proteins included nucleoside diphosphate kinase, aspartokinase, phosphoglucosamine mutase, phosphoenolpyruvate synthase, alcohol dehydrogenase GroES domain protein, NADP-dependent isocitrate dehydrogenase, DNA/RNA-binding protein Alba, 30S ribosomal protein S5, translation elongation factor 1α, thermosomes (SiRe_1214 and SiRe_1716), and von Willebrand type A (vWA) domain proteins (SiRe_1008 and SiRe_1011) (Supplementary Table S1). All the identified phosphorylated proteins were classified according to archaeal clusters of orthologous groups (arCOG) functional categories (Makarova et al., 2015). Similar to the previous phosphoproteomic analyses of *Sulfolobus* species (Esser et al., 2012; Reimann et al., 2013), the phosphorylated proteins belonged to most arCOG categories (19 out of 26 arCOG), confirming that protein phosphorylation plays a global regulatory role in archaeal cells (Supplementary Figure S4).

We found that the ratios of phosphorylated Ser, Thr, and Tyr were 65.3, 23.8, and 10.9%, respectively. The ratio of phosphorylated Tyr in our data was lower than the two previous phosphoproteomic analyses (53.6% in wild type *S. solfataricus* and 36.2−45.7% in the wild type, Δ*ptp*, or Δ*pp2a* of *S. acidocaldarius*) (Esser et al., 2012; Reimann et al., 2013). These differences may be due to the different phases of the cells used for analysis. A culture in the early log phase (OD_600_ ∼ 0.28) was used in this study, while in the two previous reports, cells of late exponential growth phase (OD_600_ ∼ 0.7 − 0.8) were used. It was reported that proteins carrying phosphorylated Tyr residues were significantly more abundant in Hela cells (Sharma et al., 2014). The lower OD_600_ of the samples in this study might have an effect on protein profiles (probably low abundant proteins), while cultures with a higher OD_600_ contained more abundant proteins that might have had more phosphorylated Tyr sites. Moreover, the relatively low level of Tyr phosphorylation could have been caused by the Tyr kinase activity versus phosphor-tyrosine phosphatase (PTP) activity (Hunter, 2009). It seems that most Tyr kinases in eukaryotes are only activated in specific circumstances for phosphorylation, whereas phosphorylated Tyr residues usually have a short half-life due to the high activity of PTPs unless they have been protected by binding to SH2 or PTP domain proteins (Sadowski et al., 1986). Thus far, it is unclear whether archaeal Tyr kinases are activated under specific conditions. However, the activity of *S. acidocaldarius* PTP was much higher than that of PP2A (Reimann et al., 2013), indicating that the half-life of Tyr phosphorylation may be too short to be significantly detected in certain stages of *Sulfolobus* cells.

### UV Treatment-Induced Pathways

For quantification of phosphorylation changes, those with more than 1.3-fold differential phosphorylation in both biological replicates were selected for detailed analysis. The use of >1.3-fold cutoff was because phosphorylated sites with >1.3-fold change comprised 5–10% of the total phosphorylated sites detected and this threshold was used in several proteomics studies in archaea (e.g., Cao et al., 2019). The phosphorylation changes might reflect the increase or decrease of steady state protein in phosphorylated state. However, the methodology used in this study could not exclude that variation in synthesis rates and/or degradation rates of proteins also lead to phosphorylation changes.

**FIGURE 3:**
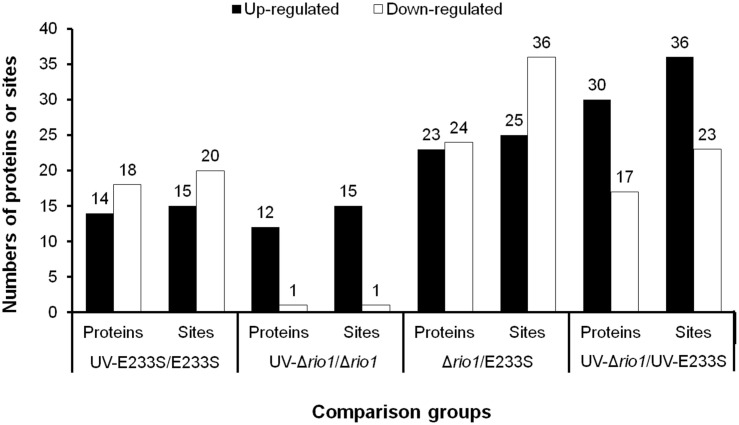
Statistics of differentially phosphorylated protein sites in various comparison groups. The numbers of phosphorylated proteins (sites) with a threshold of >1.3-fold are summarized here. The increased (up-) and decreased (down-) amount of phosphorylated proteins and sites are shown in filled and empty bars, respectively.

**FIGURE 4:**
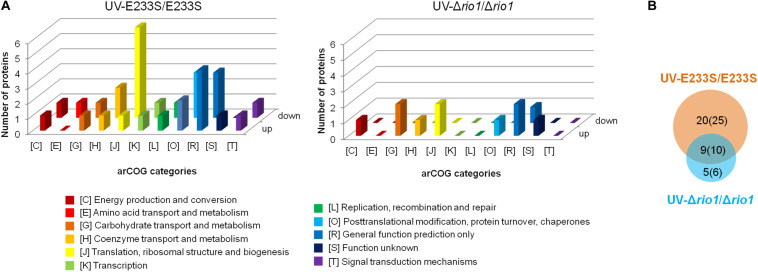
Analysis of differentially phosphorylated proteins in a comparison of UV-treated E233S versus E233S and UV-treated Δ*rio1* versus Δ*rio1*. **(A)** COG analysis of differentially phosphorylated proteins in UV-treated E233S versus E233S (left panel) and UV-treated Δ*rio1* versus Δ*rio1* (right panel). Both increased (up) and decreased (down) phosphorylated proteins are shown. The categories of arCOG are indicated at the bottom. **(B)** Overlap analysis of the numbers of differentially phosphorylated proteins (sites) between UV-treated E233S versus E233S (orange) and UV-treated Δ*rio1* versus Δ*rio1* (cyan). The numbers in brackets are those of phosphorylated sites.

To identify UV-dependent protein/site phosphorylation, the phosphorylated proteins and sites were compared between UV-treated E233S and untreated E233S. The phosphorylation of 15 sites on 14 proteins increased after UV-treatment, while the phosphorylation of 20 sites on 18 proteins decreased (Figure 3). These proteins were mainly associated with cellular metabolism (arCOG C, E, G, H); translation, ribosomal structure, and biogenesis (arCOG J); and posttranslational modification, protein turnover, and chaperones (arCOG O) (Figure 4A). This indicated that the processes of both cellular metabolism and protein synthesis changed (probably inhibited for preserving energy after DNA damage) by phosphorylation and PTMs were induced on specific proteins in response to UV irradiation.

#### Phosphorylation of Signal Transduction Proteins

Interestingly, two and three differentially phosphorylated sites were identified in the Hanks-type protein kinase (SiRe_2056, ePK1) and vWA domain-containing protein SiRe_1011 (vWA2), respectively. The phosphorylation level of ePK1 at T314 decreased by 0.59-fold, whereas that on T592 increased 2.0-fold. The phosphorylation of three residues of vWA2, S304, T314, and T327 decreased by 0.64, 0.68, and 0.74-fold, respectively (Table 1 and Supplementary Table S2). The phosphorylation of different protein sites may correlate with various regulatory functions. Protein kinases would transfer signals and vWA domain proteins may mediate protein-protein interaction (Whittaker and Hynes, 2002). Strikingly, decreased phosphorylation of ePK1 at T314 and vWA2 at S304, T314, and T327 also occurred when *rio1* was deleted (Table 1). Moreover, phosphorylation of another vWA domain-containing protein SiRe_1008 (vWA1) at T353 decreased in both UV-treated E233S cells and UV-treated Δ*rio1*, indicative of UV-dependent but Rio1-independent manner (Table 1 and Supplementary Tables S2, S3).

**TABLE 1:** Differentially phosphorylated proteins involved in signal transduction, DNA replication/repair, and translation in the four comparisons of UV-E233S/E233S, UV-Δ*rio1*/Δ*rio1*, Δ*rio1*/E233S, and UV-Δ*rio1*/UV-E233S.

Gene ID	Protein description	Residues	UV-E233S/E233S	UV-Δ*rio1*/Δ*rio1*	Δ*rio1*/E233S	UV-Δ*rio1*/UV-E233S
SiRe_2056	Eukaryotic-like Serine/threonine Protein kinase (ePK1)	T314	0.586		0.571	
		T592	1.983			0.714
SiRe_1008	Von Willebrand type A (vWA1)	T353	0.658	0.685		
SiRe_1011	Von Willebrand type A (vWA2)	S304, T314, T327	0.641, 0.677, 0.736		0.494, 0.691, 0.661	
		Y303, T351			0.637, 0.645	
SiRe_1010	FHA domain containing protein (FHA)	T97			0.660	
SiRe_1776	Elongation factor 1-α (EF1-α)	T281	0.740		1.801	2.048
		T284			1.742	1.947
		S196, S244		1.333, 1.505		
		T327				1.385
SiRe_1281	Elongation factor 2 (EF2)	S662			1.887	
SiRe_0668	DNA-binding 7 kDa protein (Sis7d)	T41		1.321		
		S47	2.451	2.872		
SiRe_0508	SMC-like protein	Y263	2.792	2.679		
		Y266	3.859	2.943	1.321	
SiRe_0161	Single-stranded DNA-binding protein (SSB)	S89			1.316	1.446
SiRe_1451	DNA polymerase (DNA pol)	T22				0.523

**FIGURE 5:**
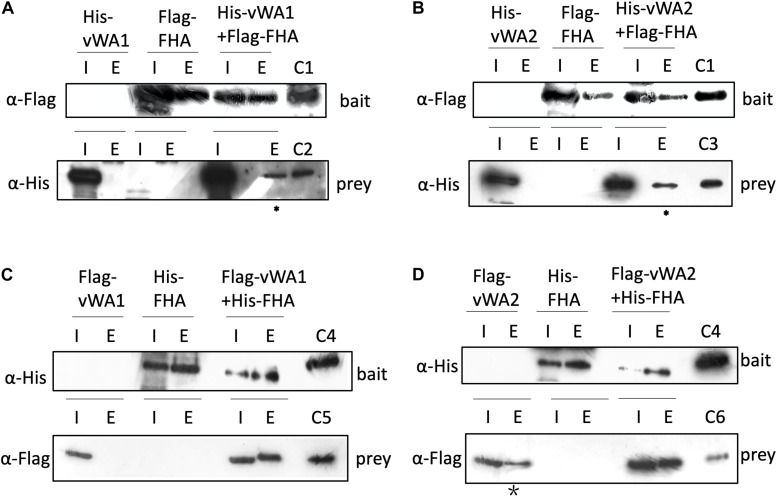
Interaction of FHA and vWAs by pull down. **(A,B)** Co-purification of His-vWA1**(A)**/vWA2**(B)** and Flag-FHA in *E. coli*. His-vWA1 (or vWA2) and Flag-FHA were co-expressed in *E. coli*. Five hundred milliliters of the *E. coli* cells were resuspended in 40 ml buffer A and protein samples were prepared as described in the section “Materials and Methods.” Total soluble protein and the enriched sample (20 μl) from approximately 40 ml supernatant (0.05%) and 9 ml elute (0.22%), respectively, were analyzed by Western blot. C1, control of Flag-FHA purified from *E. coli*; C2, His-tagged vWA1 purified from *E. coli*; C3, His-tagged vWA2 purified from *E. coli*. The asterisks represent samples concentrated by 12 times (2.6% of the total elute) before loading to the gels. **(C,D)** Co-purification of Flag-vWA1**(C)**/vWA2**(D)** and His-FHA in *S. islandicus*. One hundreds milliliters of *S. islandicus* cells harboring the co-expression plasmid were cultivated to OD600∼0.2 and protein samples were prepared as described in the section “Materials and Methods.” Total soluble protein and the enriched sample (40 μl) from approximately 10 ml supernatant (0.4%) and 2 ml elute (2.0%), respectively, were analyzed by Western blot. C4, His-tagged FHA purified from *E. coli*; C5, Flag-vWA1 purified from *S. islandicus*; C6, Flag-vWA2 purified from *S. islandicus*. The star indicates that Flag-vWA2 bound to Ni-NTA beads weakly. I, input; E, elute.

In the *S. islandicus* genome, an FHA domain-containing protein SiRe_1010 is adjacent to the two vWA domain proteins (SiRe_1008 and SiRe_1011) and its phosphorylation at T97 decreased in Δ*rio1* versus E233S (Table 1 and Supplementary Table S4). In eukaryotes, the FHA domain is often a part of large protein kinases and is involved in DDR, DNA repair, and replication (Durocher and Jackson, 2002; Mohammad and Yaffe, 2009). In bacteria, FHA-containing proteins were able to bind phosphorylated proteins (Alderwick et al., 2006; Niebisch et al., 2006). In *S. acidocaldarius*, the interaction of FHA and vWA1 was mediated by phosphorylation and the cell motility was regulated by this interaction (Reimann et al., 2012; Albers and Jarrell, 2015; Hoffmann et al., 2019; Ye et al., 2020). Our data suggested that vWA2 might also be phosphorylated and interacted with FHA *in vivo*. To test this hypothesis, co-expression of FHA and vWA1 (or vWA2) was performed in *E. coli* and *S. islandicus*. *In vivo* pull down was carried out by anti-Flag beads to obtain Flag-FHA and Western blot with His-tag specific antibody to determine the co-exist of His-vWAs in *E. coli*, or by Ni-NTA purification of His-tagged FHA and Western blot for detection of co-purified vWAs by anti-Flag antibody in *S. islandicus*. We revealed that vWA2 interacted with FHA (Figures 5B,D). Similar to *S. acidocaldarius* counterparts, vWA1 was able to interact with FHA both in *E. coli* and *S. islandicus* (Figures 5A,C). Interestingly, more proteins were pulled down in *S. islandicus* cell*s* than in *E. coli.* We assume that PTM including phosphorylation enhances the protein interaction. Consistently, a recent *in vitro* study showed that phosphorylation of *S. acidocaldarius* vWA1 stimulated formation of FHA-vWA complex, while dephosphoryation by PP2A resulted in dissociation of the complex (Ye et al., 2020). Our results indicate that phosphorylation of vWA2 may be also involved in signal transduction together with FHA. Since *S. acidocaldarius* Δ*rio1* are less motile (Hoffmann et al., 2017) and our data showed that phosphorylation of FHA and vWA2 was decreased when *rio1* was deleted, it is also possible that Rio1 might be involved in cell motility by regulating FHA/vWAs phosphorylation. Further study is needed to investigate whether and how the interactions between FHA and vWA1/vWA2 are regulated, and if these interactions play a broad role in stress responses.

#### Phosphorylation of Chromosomal Proteins

**FIGURE 6:**
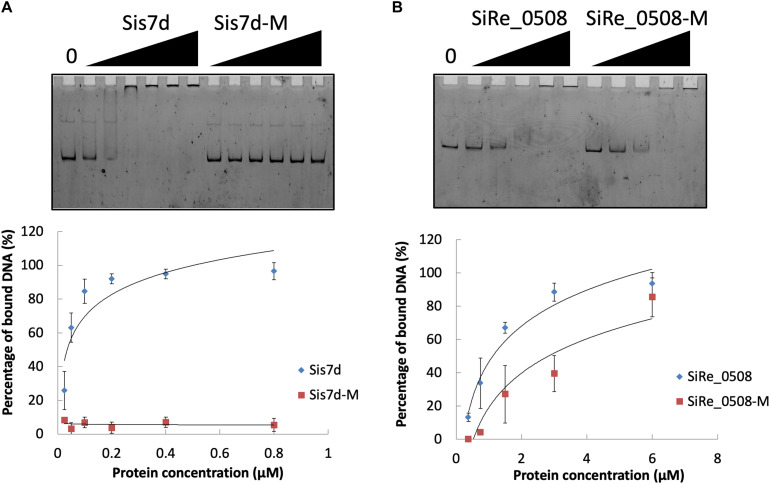
DNA binding ability of chromosome remodeling proteins and their phosphor-mimic mutants. **(A)** EMSA of Sis7d and its mutants Sis7d-T41E/S47E. The reaction was performed at 37°C for 30 min and analyzed in 8% native PAGE (see section “Materials and Methods”). Protein concentration of 0.025, 0.05, 0.1, 0.2, 0.4, and 0.8 μM was used. **(B)** EMSA of SiRe_0508 and SiRe_0508-Y263E/Y266E. Protein concentration of 0.38, 0.75, 1.5, 3, and 6 μM was used. Representative gels are shown. The quantitative data was obtained from at least three independent experiments. Error bars indicate standard deviation.

We found that the phosphorylation levels of two chromosomal proteins changed with UV irradiation. While previous report showed that the transcription of the chromatin protein Sso7d was repressed upon UV irradiation (Frols et al., 2007; Gotz et al., 2007), the phosphorylation level at T41 and S47 of the counterpart in *S. islandicus*, Sis7d, increased in our study (Table 2). Sso7d is able to bind DNA so as to increase the thermal stability of packed DNA (Robinson et al., 1998; White and Bell, 2002). Decreased Sso7d/Sis7d level would allow chromatin to be more flexible for DNA repair. T41 and S47 are located at a conserved core loop close to the DNA backbone (41, 42). Therefore, phosphorylation of Sis7d may reduce its DNA binding ability by introducing negative charge. To test the effect of phosphorylation on Sis7d, the wild type and phosphor-mimic mutant proteins (T41E/S47E) were purified and their DNA binding activities were assayed. As expected, DNA binding ability of mutant Sis7d decreased compared with the wild type protein (Figure 6A). In addition, we found that the phosphorylation of an SMC (Structural Maintenance of Chromosome) domain-containing protein (SiRe_0508) at Y263 and Y266 increased after UV treatment (Table 2). Since the SMC family proteins are usually involved in chromosome organization (Diebold-Durand et al., 2017; Wang et al., 2017), SiRe_0508 phosphorylation may inhibit its DNA affinity to impede chromosome remodeling and facilitate DNA repair. As predicted, EMSA revealed that phosphor-mimic mutant SiRe_0508-Y263E/Y266E exhibited lower dsDNA affinity than the wild type enzyme (Figure 6B). Exactly how the phosphorylation of SiRe_0508 regulates its function needs further investigation.

**TABLE 2:** Differentially phosphorylated proteins in UV-E233S/E233S and/or UV-Δ*rio1*/Δ*rio1.*

Gene ID*	Amino acid (Position)	Fold change	Protein description
SiRe_0330	S119	1.900, 2.242	FAD-dependent pyridine nucleotide-disulphideoxido reductase
SiRe_0451	S144	1.375	ATPase-like protein
SiRe_0508	Y263, Y266	2.792, 3.859; 2.679, 2.943	SMC-like protein
SiRe_0668	S47	2.451, 2.872	DNA-binding 7 kDa protein
SiRe_0668	T41	1.321	DNA-binding 7 kDa protein
SiRe_1005	S738	0.738	Aconitate hydratase 1
SiRe_1005	S664	1.345	Aconitate hydratase 1
SiRe_1008	T353	0.658, 0.685	von Willebrand factor type A
SiRe_1011	S304, T314, T327	0.641, 0.677, 0.736	von Willebrand factor type A
SiRe_1024	T6	0.368	Amino acid permease-associated region
SiRe_1033	T257	0.651	Biotin/lipoate A/B protein ligase
SiRe_1169	S35	0.679	Uncharacterized protein
SiRe_1186	S385	1.310	Valyl-tRNA synthetase
SiRe_1199	S483	1.662, 1.537	Phosphoenolpyruvate synthase
SiRe_1202	S166	2.178, 2.149	UbiD family decarboxylase
SiRe_1214	S150	0.690	Thermosome
SiRe_1214	Y351	1.810	Thermosome
SiRe_1264	S207	0.736	30S ribosomal protein S3Ae
SiRe_1274	S94	0.605	Exosome complex component Rrp41
SiRe_1304	S73	0.720	50S ribosomal protein L6
SiRe_1441	T332	1.432	V-type ATP synthase alpha chain
SiRe_1582	T334	0.638	AAA family ATPase, CDC48 subfamily
SiRe_1612	S472	1.481	Glycosylated S-layer protein, SlaA
SiRe_1716	Y310	1.417, 1.526	Thermosome
SiRe_1716	S325	0.524	Thermosome
SiRe_1776	T281	0.740	Elongation factor 1-alpha
SiRe_1776	S196, S244	1.333, 1.505	Elongation factor 1-alpha
SiRe_1800	S97	0.632	Phosphoglucosamine mutase
SiRe_1800	S97	1.390	Phosphoglucosamine mutase
SiRe_1851	T204	0.703	Lysine biosynthesis enzyme LysX
SiRe_1898	T226	1.531, 2.253	UPF0173 metal-dependent hydrolase SiRe_1898
SiRe_1900	T286	2.727, 1.323	Uncharacterized protein
SiRe_1905	S2	0.711	50S ribosomal protein L7Ae
SiRe_1920	T88	1.376	30S ribosomal protein S11
SiRe_2056	T314	0.586	Serine/threonine protein kinase
SiRe_2056	T592	1.983	Serine/threonine protein kinase
SiRe_2627	T72	0.698	VapB-type antitoxin

*Proteins in red are identified from both UV-E233S/E233S and UV-Δ*rio1*/Δ*rio1*; proteins in blue are only from UV-Δ*rio1*/Δ*rio1*; other proteins are only from UV-E233S/E233S.

#### Phosphorylation of DNA Repair/Replication Proteins

Nucleotide excision repair (NER), base excision repair (BER), mismatch repair (MMR), and homologous recombination repair are supposed to repair UV-induced DNA lesions (Courcelle and Hanawalt, 2001; Goosen and Moolenaar, 2008). In the current study, phosphorylation of multiple DNA replication/repair proteins were identified, including DnaG, DNA Ligase, replicative DNA polymerase B (PolB1, SiRe_1451), DNA topoisomerase, RFC, RadA, SSB, Hjm, XPB1, and XPD (Supplementary Table S1), although the phosphorylation levels of DNA repair proteins altered not always significantly. However, phosphorylation levels of some of the proteins changed 1.2–1.3 times or more in at least one of the biological replicates, such as PolB1, DNA topoisomerase, RadA, and XPB1. Proteins involved in the NER, BER, and MMR pathways have been shown to change significantly in their phosphorylation patterns at 6 h after UV treatment in Hela cells (Edifizi et al., 2017; Xu et al., 2017). This indicated that phosphorylation of DNA replication and repair proteins also changes in response to UV treatment in archaeal cells but to a lesser degree compared with eukaryotes. In addition, we could not rule out that the phosphorylation change of DNA repair proteins may become more apparent at the later stages of response to UV irradiation.

### Rio1 Mediated Protein Metabolism After UV Irradiation

To investigate which pathways induced by UV irradiation were dependent on Rio1, the phosphorylation changes between UV-treated Δ*rio1* cells and untreated Δ*rio1* cells (UV-Δ*rio1*/Δ*rio1*) were compared with those between UV-treated E233S cells with untreated E233S cells (UV-E233S/E233S). Our results revealed that phosphorylation of 15 sites on 12 proteins increased in UV-Δ*rio1*/Δ*rio1* (9 sites overlapped with UV-E233S/E233S), while that of only one site decreased in UV-Δ*rio1*/Δ*rio1*, which was much fewer than the number of decreased phosphorylated sites (20) from a comparison of UV-E233S/E233S (Figures 3, 4B). This suggested that Rio1 was responsible for the reduction (>1.3-fold) of most protein phosphorylation induced by UV irradiation (Figure 4A and Supplementary Table S3). We identified 25 sites on 21 proteins with >1.3-fold differential phosphorylation only in UV-treated E233S cells versus E233S, among which 6 sites on 6 proteins exhibited increased phosphorylation, and 19 sites on 17 proteins had lower phosphorylation levels (Figure 4B and Table 2). In addition, there were 10 sites (9 increased and 1 decreased) on 9 proteins that changed in both groups, representing those phosphorylation events induced by UV but independent of Rio1 (Table 2).

Among the proteins with decreased phosphorylation levels identified only in UV-treated E233S, the majority were associated with translation, ribosomal structure, and biogenesis (arCOG J, including TEF1α, the exosome complex component Rrp41, and three ribosomal proteins) and posttranslational modification, protein turnover, and chaperones (arCOG O, including ePK1, CDC48, and two thermosomes, Figure 3A and Table 2). These indicated that Rio1 mediated protein metabolism after UV irradiation in an indirect manner. In a study of *Caenorhabditis elegans*, the genes involved in protein synthesis, refolding, and degradation were transcriptionally reduced upon UV-induced DNA damage, suggesting that protein recycling was impaired due to general energy depletion (Edifizi et al., 2017). Our data revealed that these processes were also regulated by phosphorylation after UV treatment, probably by inhibition. It is unknown whether the inhibition of the phosphorylation by Rio1 was due to inactivation of a second protein kinase or enhancement of protein phosphatase. However, we cannot exclude that Rio1 might directly interact with these proteins for inhibition, since the interaction of yeast Rio1 with ribosomal proteins, exosome complex components, and chaperones has been detected by yeast two-hybrid (Iacovella et al., 2018). Moreover, in eukaryotes, Rio1 is a subunit of the pre-40S ribosome and is involved in pre-rRNA processing and small ribosomal subunit maturation via a direct interaction with other pre-40S subunits (Ferreira-Cerca et al., 2014; Turowski et al., 2014; Ameismeier et al., 2018). Most likely, one of the Rio1 roles in response to UV irradiation was inhibition of protein synthesis and turnover to reduce energy consuming.

Several proteins exhibited increased phosphorylation levels in UV-treated E233S but not in UV-treated Δ*rio1*, among which were ePK1 (SiRe_2056), the S-layer protein SlaA (SiRe_1612), and a thermosome (SiRe_1214). Our *in vitro* kinase assay showed that Rio1 was able to phosphorylate ePK1 (Supplementary Figure S5), conversely, ePK1 could also phosphorylate Rio1 as revealed previously (Huang et al., 2017). The detailed regulatory mechanism between these kinases needs further investigation. It will be very interesting to determine whether ePK1 and SlaA are potential Rio1 substrates *in vivo* and what roles they play in DDR.

Remarkably, PolB1 phosphorylation decreased in UV-treated Δ*rio1* cells as compared to UV-treated E233S cells but not significantly different (<1.3-fold) in Δ*rio1* cells compared to E233S cells, implying that the phosphorylation change were induced by UV and amplified by Rio1, affecting (probably inhibiting) DNA replication (Table 1 and Supplementary Table S1).

Previously, we found that Rio1 phosphorylated the Holliday junction resolvase Hjc, which inhibited its catalytic activity and increased the cell viability in the presence of multiple DNA lesions. We proposed a regulatory role for Rio1 in stalled DNA replication fork resolution (Huang et al., 2019). Phosphorylation of Hjc was not identified in this study, probably due to low protein level, which could become even lower since *hjc* was transcriptionally down-regulated after UV treatment in *S. acidocaldarius* (13). Similarly, we did not identify phosphorylation of Orc1-2 or Tfb3 in a recently identified Orc1-2 dependent DDR network in *Sulfolobus* (Feng et al., 2018; Schult et al., 2018; Sun et al., 2018). The reason could also be due to low abundance of phosphorylated proteins at early stage of UV treatment. In this network, the Orc1-2 is up-regulated in the presence of DNA damage agents NQO or UV. The protein binds to the promoter regions of the gene itself and DDR genes and activates the expression of the Orc1-2, transcription factor TFB3, proteins involved in DNA synthesis (Dpo2), the *ups* operon and the *ced* gene transfer system (Ajon et al., 2011; van Wolferen et al., 2016), and represses the expression of genes involved in DNA replication initiation, genome segregation, and cell division (Sun et al., 2018). Since we did not find a conserved DNA motif for Orc1-2 binding to the DDR gene promoters (5′-ANTTTC-3′) in the *rio1* promoter region (data not shown), which was bound by Orc1-2 for regulation (Le et al., 2017; Sun et al., 2018), Rio1-mediated network might be independent of the Orc1-2-centered network. In addition, Rio1 was induced earlier than that of Orc1-2 after UV-treatment (peak at 30 min versus 90 min) according to a previous transcriptomic study (Gotz et al., 2007), suggesting that the expression of *rio1* after UV irradiation may not in fact be induced by Orc1-2 and the Rio1-mediated network might be independent of the Orc1-2-centered network. Whether and how Rio1 interplays with the Orc1-2- centered network need further investigation.

### *rio1* Deletion Resulted in Phosphorylation Changes in Multiple Cellular Metabolic Pathways

The comparison of Δ*rio1* cells versus E233S cells revealed that phosphorylation of 25 sites on 23 proteins increased, while that at 36 sites on 24 proteins decreased (Figure 3 and Supplementary Table S4). The arCOG categories of these proteins revealed that more proteins associated with various cellular metabolisms (arCOG C, E, G, H) appeared in Δ*rio1* cells versus E233S compared with UV-treated E233S versus untreated E233S cells (Supplementary Figure S6), suggesting that Rio1 also regulates metabolism without DNA damage treatment in addition to protein synthesis/turnover.

**FIGURE 7:**
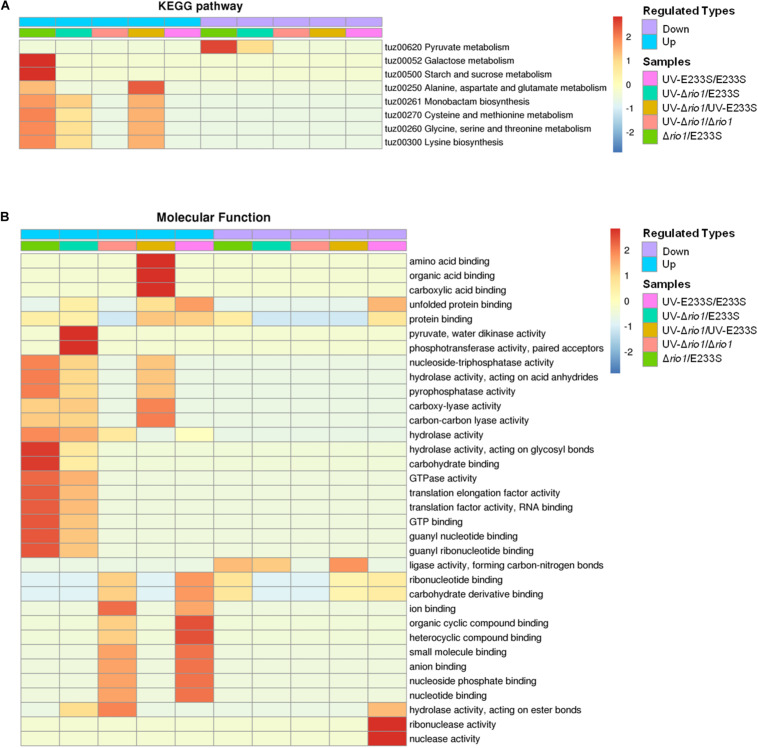
Functional enrichment of the five comparison groups. **(A)** KEGG pathway. **(B)** Molecular function. Both increase (up) and decrease (down) of differentially phosphorylated proteins involved in processes or pathways are shown. Red to blue represents the enrichment strength in log10 values.

To further investigate the impact of differentially phosphorylated proteins (>1.3-fold) on cellular physiological processes, the functional enrichment including KEGG pathway and GO categories was analyzed for each comparison group. For KEGG enrichment, multiple proteins with increased phosphorylation were enriched in Δ*rio1*. These proteins are involved in various metabolic pathways, including galactose metabolism, starch and sucrose metabolism, alanine, aspartate, and glutamate metabolism, monobactam biosynthesis, cysteine and methionine metabolism, glycine, serine, and threonine metabolism, and lysine biosynthesis (Figure 7A). The latter five metabolic pathways were also enriched in the UV-treated Δ*rio1* versus UV-treated E233S cells, confirming the important roles of Rio1 in cellular metabolism independent of UV irradiation (Figure 7A and Supplementary Table S5). As Δ*rio1* grew faster than wild type, Rio1 may regulate growth rate via protein synthetic capacity and/or cellular metabolism.

In addition, GO enrichment based on molecular function showed that *rio1* deletion mainly led to increased phosphorylation of proteins with lyase or hydrolase activities, whereas UV irradiation resulted in increased phosphorylation of those with ion or small molecular binding activities, suggesting that these two treatments (*rio1* deletion and UV irradiation) led to phosphorylation changes of proteins with different activities for different downstream reactions (Figure 7B).

Remarkably, the phosphorylation of three Pur proteins (SiRe_1379/PurL, SiRe_1382/PurC, and SiRe_1753/PurA) decreased in both Δ*rio1* cells compared to E233S cells and UV-treated Δ*rio1* cells compared to UV-treated E233S (Supplementary Tables S4, S5), but not in UV-E233S/E233S, indicating the phosphorylation change is Rio1-dependent but not UV-induced. It has been shown that the gene cluster of nucleotide (purine) biosynthesis (*purC/Saci_1607, purS*/*Saci_1608*, and *purL/Saci_1610*) and a putative purine transporter gene (*Saci_0214*) were transcriptionally down-regulated in response to UV in *S. acidocaldarius* (Hoffmann et al., 2017; Schult et al., 2018). These changes suggested that nucleotide synthesis and uptake were both repressed after UV treatment, probably for the purpose of slowing down DNA replication. Consistent with this, the transcripts of the gene cluster (*Saci_1607-Saci_1613*) and the uptake gene (*Saci_0214*) have been shown to be higher in S-phase, where a large number of DNA replication events occur (Lundgren and Bernander, 2007). Our results suggest that purine biosynthesis is regulated probably by Rio1-mediated phosphorylation. Strikingly also, phosphorylation of SSB increased after *rio1* deletion regardless of UV-treatment (Table 1). Phosphorylation of these proteins could provide another layer of regulation, although it was in a UV-independent manner in certain circumstances.

### Identification of Phosphorylation Motifs

**FIGURE 8:**
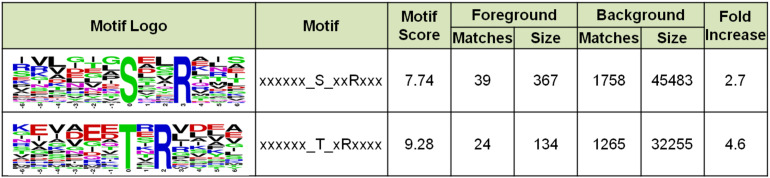
Motif enrichment analysis of the identified phosphorylation Ser and Thr sites in this study. Ser and Thr at specific positions in modified-13-mers (6 amino acids upstream and downstream of phosphorylation site) for all identified protein sequences were collected, and all the database protein sequences were used as the background database parameter. The software package MoMo (motif-x algorithm) was used for motif enrichment analysis. The minimum number of occurrences was set to 20.

To understand whether *Sulfolobus* phosphorylation has a bias toward any amino acid motif, we analyzed the amino acid composition of the identified phosphorylation sites using software MoMo. The frequencies of 6 amino acids surrounding all phosphorylation sites were summarized, and the result showed that Arg appeared at the +3 position of 39 out of 367 phosphorylated Ser sites, which was enriched by 2.7-fold compared with the background of Arg containing peptides from all identified peptides (Figure 8). In addition, Arg was enriched at the +2 position of phosphorylated Thr by 4.6-fold (Figure 8). These results indicate that the positively charged amino acid Arg, which frequently appeared at the +2/3 position downstream of phosphorylated sites, might facilitate the phosphorylation reaction of certain *Sulfolobus* protein kinases.

Our study revealed a complex network and a number of putative target proteins of Rio1 in early response to DNA damage. Many of the phenomena need further verification and some are worthy further exploration. Nevertheless, *Saccharomyces cerevisiae* Rio1 was shown to interact with a number of factors involved in ribosome production, protein synthesis and turnover, metabolism, energy production, and cell cycle progress by yeast two-hybrid screen (Iacovella et al., 2018), suggesting that there may be conserved function and mechanism of Rio1 proteins from eukaryotes and archaea. Further study on which subset of proteins is regulated by Rio1-dependent phosphorylation and whether archaeal Rio1 binds certain gene promoters directly for regulation, similar to yeast Rio1, will also be needed. Furthermore, it will be interesting to study on how Rio1 is regulated in response to DNA damage and other stresses.

## Data Availability Statement

The datasets generated for this study can be found in the ProteomeXchange, accession: PXD021663. Project Webpage: http://www.ebi.ac.uk/pride/archive/projects/PXD021663.

## Author Contributions

QH designed the experiments, conducted most of the experiments, analyzed the data, and wrote the manuscript. ZL performed the colony formation assay and *in vivo* pull down. PW conducted the strain cultivation experiments. JN helped to analyze the data and revised the manuscript. YS conceived the project and revised the manuscript. All the authors approved the final version of the manuscript.

## Conflict of Interest

The authors declare that the research was conducted in the absence of any commercial or financial relationships that could be construed as a potential conflict of interest.

## Footnotes

1 http://www.ebi.ac.uk/GOA/

2 http://www.ebi.ac.uk/interpro/

## Abbreviations

PTM: post-translational modification
DDR: DNA damage response
UV: ultraviolet
TFB3: transcription factor B3
Rio: right open reading frame
MCS: multiple cloning site
TMT: tandem mass tags
SCX: strong cation exchange
IMAC: immobilized metal affinity chromatography
PBS: phosphate-buffered saline
LC-MS/MS: liquid chromatography tandem mass spectrometry
Q-TOF: quadrupole time-of-flight
GO: gene ontology
KEGG: Kyoto Encyclopedia of Genes and Genomes
arCOG: archaeal clusters of orthologous groups
RSD: relative standard deviation
vWA: von Willebrand type A
TEF: translation elongation factor
PTP: phosphotyrosine phosphatases
SMC: structural maintenance of chromosome.
