# Supplementary material for: Phosphoproteomic Analysis Reveals Rio1-Related Protein Phosphorylation Changes in Response to UV Irradiation in Sulfolobus islandicus REY15A
Source: Front Microbiol. 2020 Dec 3;11:586025. doi: 10.3389/fmicb.2020.586025 (PMC7744417; doi:10.3389/fmicb.2020.586025)
Supplement: Supplementary file 1 [file Presentation_1.PPTX]

## Slide 1
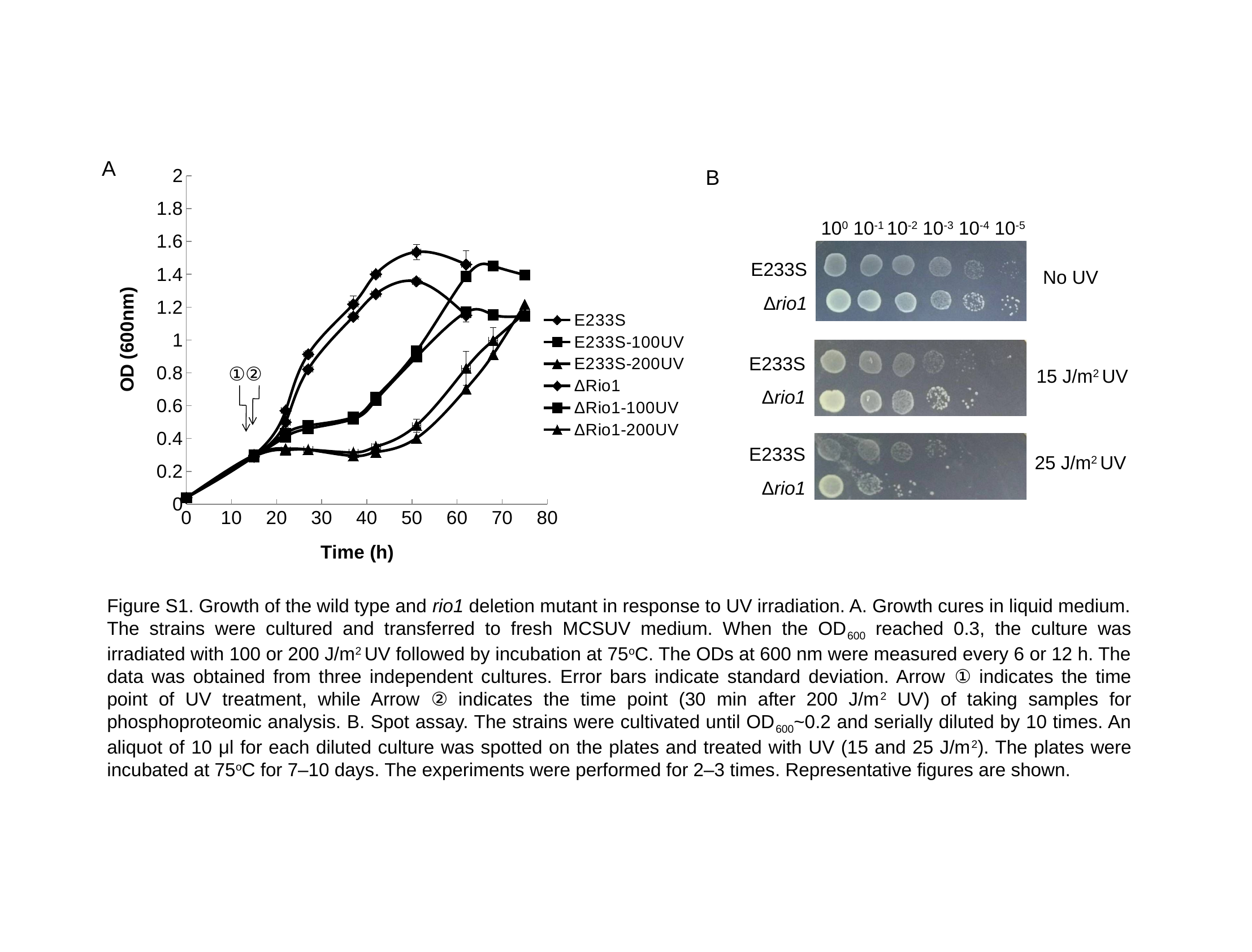

A
### Chart
| Category | E233S | E233S-100UV | E233S-200UV | ΔRio1 | ΔRio1-100UV | ΔRio1-200UV |
|---|---|---|---|---|---|---|B
100 10-1 10-2 10-3 10-4 10-5
E233S Δrio1
No UV
E233S Δrio1
①②
15 J/m2 UV
E233S Δrio1
25 J/m2 UV
Figure S1. Growth of the wild type and rio1 deletion mutant in response to UV irradiation. A. Growth cures in liquid medium. The strains were cultured and transferred to fresh MCSUV medium. When the OD600 reached 0.3, the culture was irradiated with 100 or 200 J/m2 UV followed by incubation at 75oC. The ODs at 600 nm were measured every 6 or 12 h. The data was obtained from three independent cultures. Error bars indicate standard deviation. Arrow ① indicates the time point of UV treatment, while Arrow ② indicates the time point (30 min after 200 J/m2 UV) of taking samples for phosphoproteomic analysis. B. Spot assay. The strains were cultivated until OD600~0.2 and serially diluted by 10 times. An aliquot of 10 μl for each diluted culture was spotted on the plates and treated with UV (15 and 25 J/m2). The plates were incubated at 75oC for 7–10 days. The experiments were performed for 2–3 times. Representative figures are shown.

## Slide 2
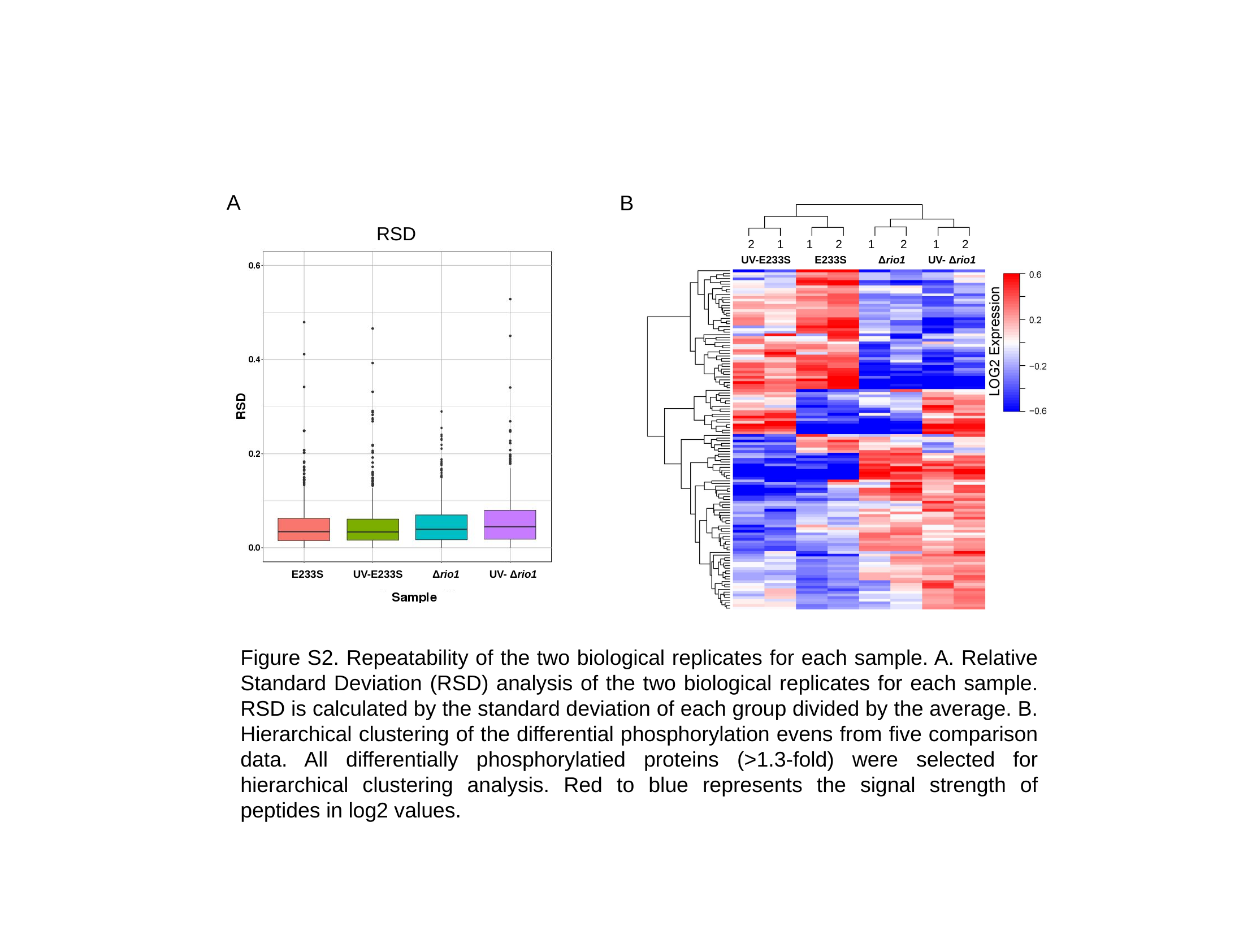

A
B
RSD
2 1 1 2 1 2 1 2
Δrio1
UV- Δrio1
UV-E233S
E233S
E233S UV-E233S Δrio1 UV- Δrio1
Figure S2. Repeatability of the two biological replicates for each sample. A. Relative Standard Deviation (RSD) analysis of the two biological replicates for each sample. RSD is calculated by the standard deviation of each group divided by the average. B. Hierarchical clustering of the differential phosphorylation evens from five comparison data. All differentially phosphorylatied proteins (>1.3-fold) were selected for hierarchical clustering analysis. Red to blue represents the signal strength of peptides in log2 values.

## Slide 3
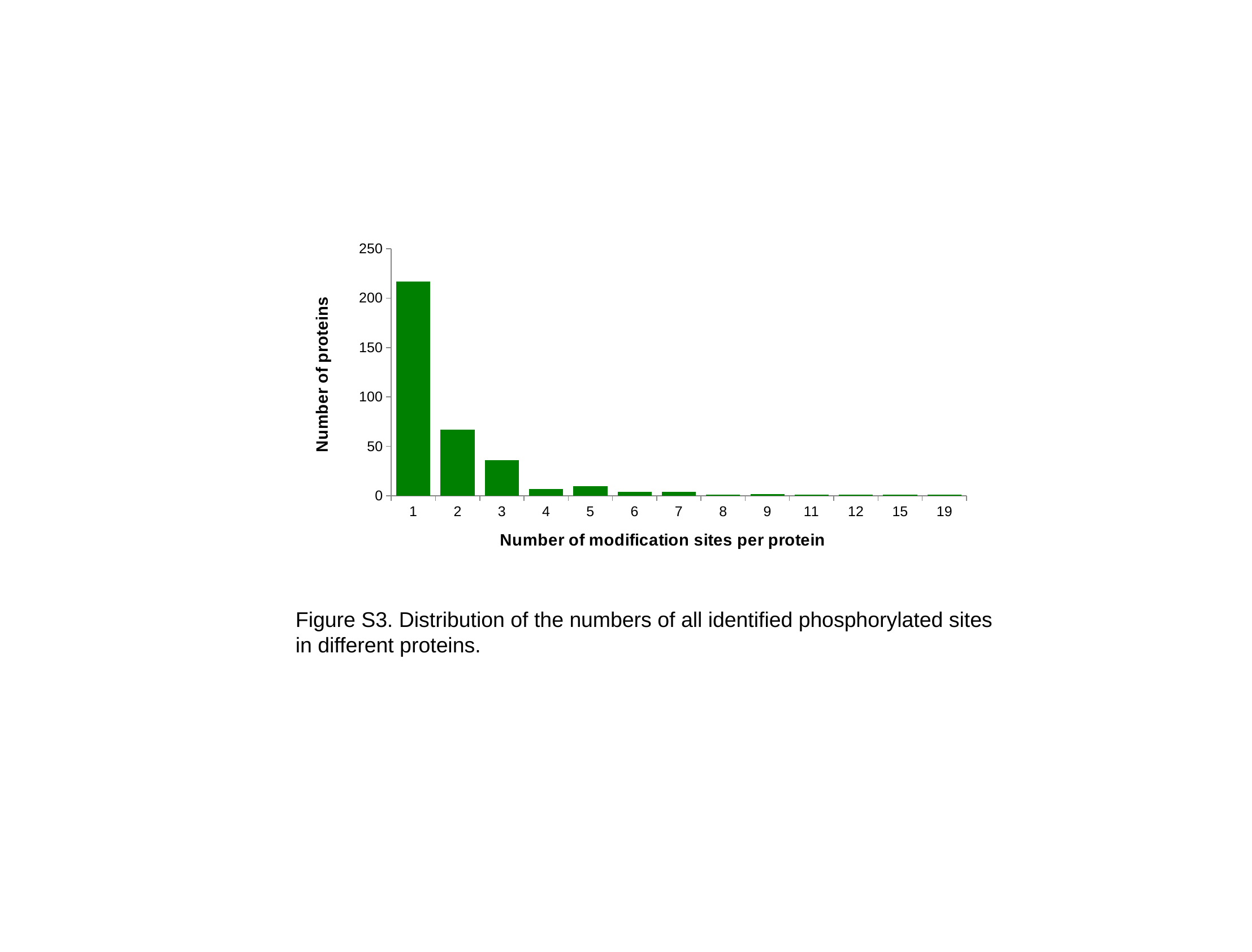

### Chart
| Category | |
|---|---|
| 1 | 217.0 |
| 2 | 67.0 |
| 3 | 36.0 |
| 4 | 7.0 |
| 5 | 10.0 |
| 6 | 4.0 |
| 7 | 4.0 |
| 8 | 1.0 |
| 9 | 2.0 |
| 11 | 1.0 |
| 12 | 1.0 |
| 15 | 1.0 |
| 19 | 1.0 |Figure S3. Distribution of the numbers of all identified phosphorylated sites in different proteins.

## Slide 4
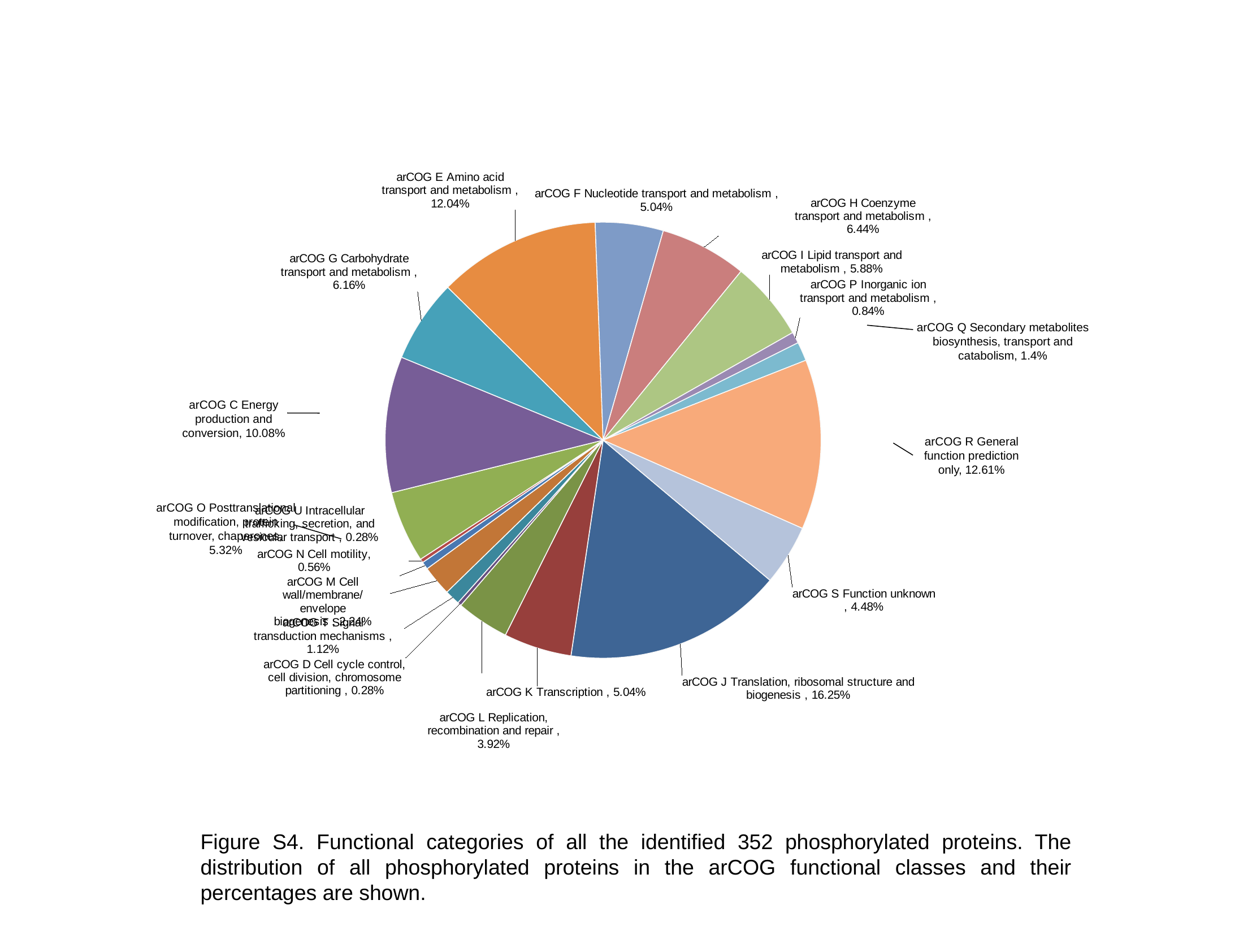

### Chart
| Category | No. of Protein |
|---|---|
| arCOG J Translation, ribosomal structure and biogenesis | 58.0 |
| arCOG K Transcription | 18.0 |
| arCOG L Replication, recombination and repair | 14.0 |
| arCOG D Cell cycle control, cell division, chromosome partitioning | 1.0 |
| arCOG T Signal transduction mechanisms | 4.0 |
| arCOG M Cell wall/membrane/envelope biogenesis | 8.0 |
| arCOG N Cell motility | 2.0 |
| arCOG U Intracellular trafficking, secretion, and vesicular transport | 1.0 |
| arCOG O Posttranslational modification, protein turnover, chaperones | 19.0 |
| arCOG C Energy production and conversion | 36.0 |
| arCOG G Carbohydrate transport and metabolism | 22.0 |
| arCOG E Amino acid transport and metabolism | 43.0 |
| arCOG F Nucleotide transport and metabolism | 18.0 |
| arCOG H Coenzyme transport and metabolism | 23.0 |
| arCOG I Lipid transport and metabolism | 21.0 |
| arCOG P Inorganic ion transport and metabolism | 3.0 |
| arCOG Q Secondary metabolites biosynthesis, transport and catabolism | 5.0 |
| arCOG R General function prediction only | 45.0 |
| arCOG S Function unknown | 16.0 |
arCOG Q Secondary metabolites biosynthesis, transport and catabolism, 1.4%
arCOG C Energy production and conversion, 10.08%
arCOG R General function prediction only, 12.61%
arCOG O Posttranslational modification, protein turnover, chaperones, 5.32%
Figure S4. Functional categories of all the identified 352 phosphorylated proteins. The distribution of all phosphorylated proteins in the arCOG functional classes and their percentages are shown.

## Slide 5
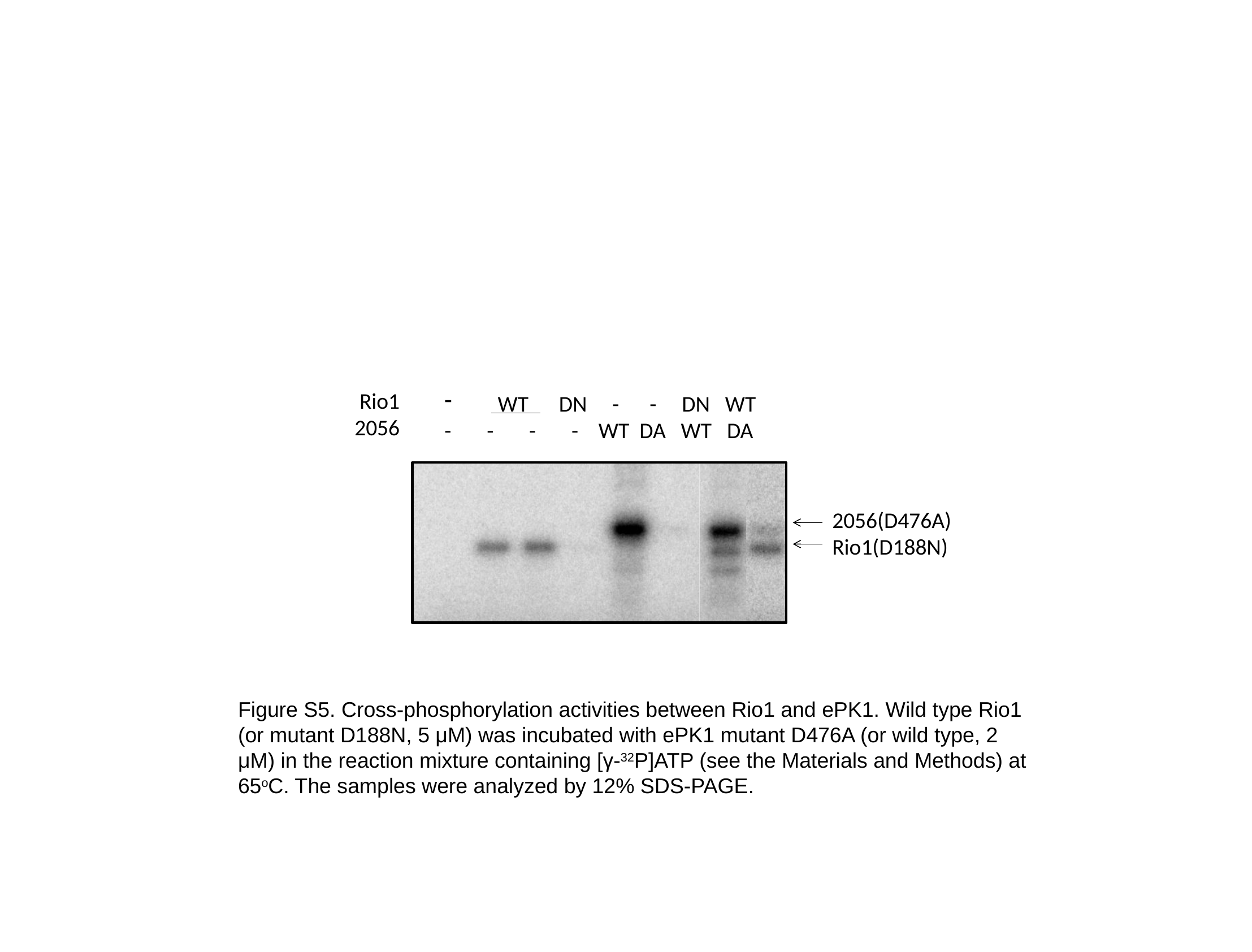

#
Rio1
2056
 WT DN - - DN WT - - - - WT DA WT DA
2056(D476A)
Rio1(D188N)
Figure S5. Cross-phosphorylation activities between Rio1 and ePK1. Wild type Rio1 (or mutant D188N, 5 μM) was incubated with ePK1 mutant D476A (or wild type, 2 μM) in the reaction mixture containing [γ-32P]ATP (see the Materials and Methods) at 65oC. The samples were analyzed by 12% SDS-PAGE.

## Slide 6
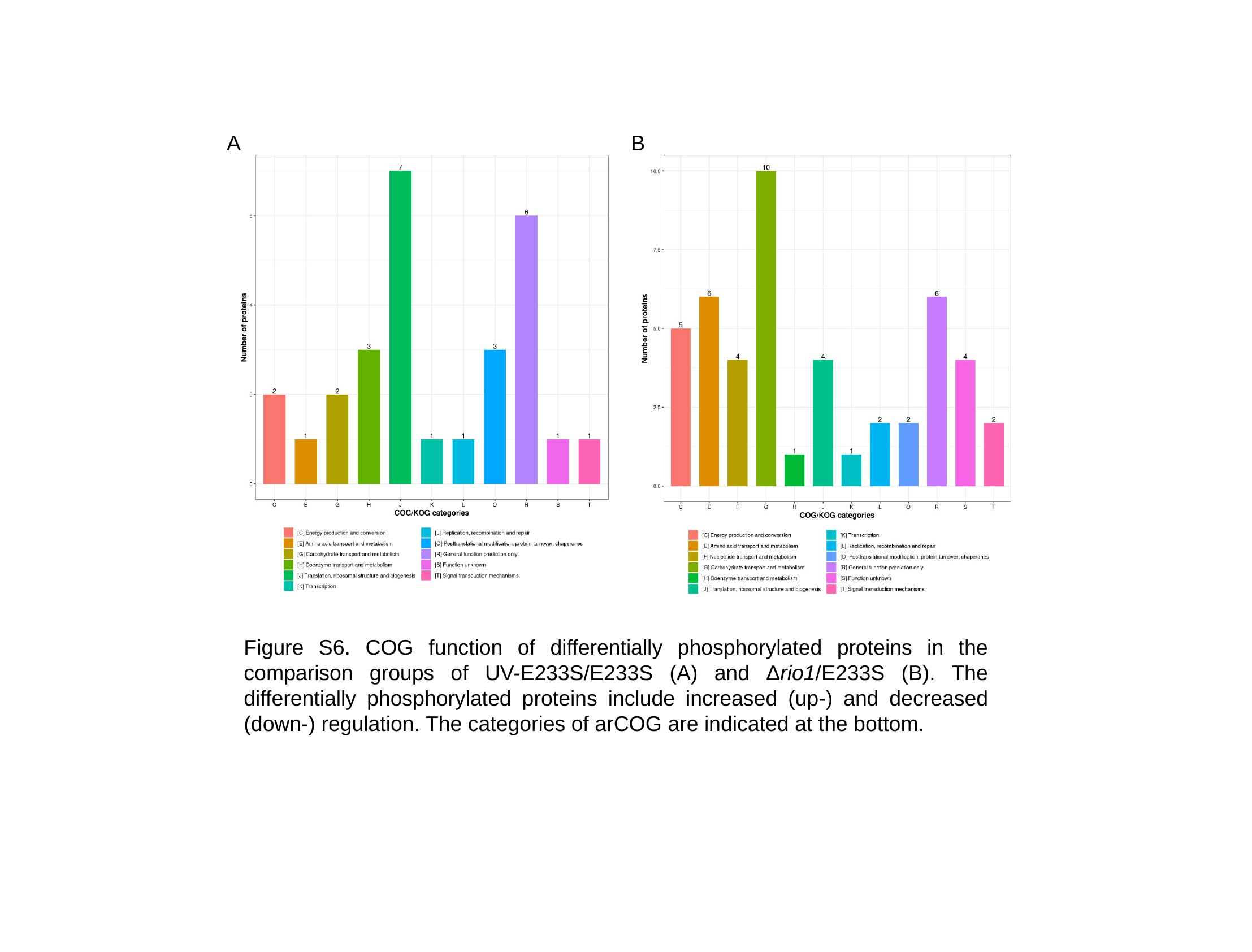

A
B
Figure S6. COG function of differentially phosphorylated proteins in the comparison groups of UV-E233S/E233S (A) and Δrio1/E233S (B). The differentially phosphorylated proteins include increased (up-) and decreased (down-) regulation. The categories of arCOG are indicated at the bottom.
